# 

**Published:** 1877-11

**Authors:** 


					﻿Vol. 24.
NOVEMBER, 1877.
No. 11.
TWENTY-FOURTH YEAR.
HALL’S
Journal of Health.
PUBLISHED AT BIBLE HOUSE,
THIRD AVENUE, CORNER OF NINTH STREET,
E. H. GIBBS, M.D., Editor.
AGENTS FOR THE TRADE:
AMERICAN NEWS COMPANY, 121 NASSAU STREET.
NEW YORK NEWS COMPANY, 18 BEEKMAN STREET.
Terms, $1.50 a Year.
SINGLE NUMBER, 15 CENTS.
K«mt & Co.. Printers, 11 Frankfort St., Now York.
				

